# The Genomic and Biologic Landscapes of Breast Cancer and Racial Differences

**DOI:** 10.3390/ijms252313165

**Published:** 2024-12-07

**Authors:** Sapthala P Loku Galappaththi, Kelly R. Smith, Enas S. Alsatari, Rachel Hunter, Donna L. Dyess, Elba A. Turbat-Herrera, Santanu Dasgupta

**Affiliations:** 1 Department of Pathology, Frederick P. Whiddon College of Medicine, University of South Alabama, Mobile, AL 36604, USA; sl1822@jagmail.southalabama.edu (S.P.L.G.); krs808@jagmail.southalabama.edu (K.R.S.); esa2222@jagmail.southalabama.edu (E.S.A.); etherrera@health.southalabama.edu (E.A.T.-H.); 2 Mitchell Cancer Institute, University of South Alabama, Mobile, AL 36688, USA; 3 Department of Surgery, Frederick P. Whiddon College of Medicine, University of South Alabama, Mobile, AL 36688, USAldyess@health.southalabama.edu (D.L.D.); 4 Department of Biochemistry and Molecular Biology, Frederick P. Whiddon College of Medicine, University of South Alabama, Mobile, AL 36688, USA

**Keywords:** mtDNA mutations, nuclear DNA mutations, microbiome, racial differences, breast cancer

## Abstract

Breast cancer is a significant health challenge worldwide and is the most frequently diagnosed cancer among women globally. This review provides a comprehensive overview of breast cancer biology, genomics, and microbial dysbiosis, focusing on its various subtypes and racial differences. Breast cancer is primarily classified into carcinomas and sarcomas, with carcinomas constituting most cases. Epidemiology and breast cancer risk factors are important for public health intervention. Staging and grading, based on the TNM and Nottingham grading systems, respectively, are crucial to determining the clinical outcome and treatment decisions. Histopathological subtypes include in situ and invasive carcinomas, such as invasive ductal carcinoma (IDC) and invasive lobular carcinoma (ILC). The review explores molecular subtypes, including Luminal A, Luminal B, Basal-like (Triple Negative), and HER2-enriched, and delves into breast cancer’s histological and molecular progression patterns. Recent research findings related to nuclear and mitochondrial genetic alterations, epigenetic reprogramming, and the role of microbiome dysbiosis in breast cancer and racial differences are also reported. The review also provides an update on breast cancer’s current diagnostics and treatment modalities.

## 1. Introduction

Cancer remains a major health concern globally, with breast cancer being the most frequently diagnosed cancer among women [1]. Breast cancer is an extensively heterogeneous disease that originates in the epithelium or myoepithelium in the breast tissues and can vary widely in its histopathology, clinical behavior, and response to treatment. This review aims to provide a comprehensive overview of breast cancer, focusing on genomics, biology and the microbiome niche. Breast cancer can be classified into several types, primarily carcinoma and sarcoma. Carcinomas, which constitute most breast cancer cases, originate from the epithelial cells lining the ducts and lobules. These include invasive ductal carcinoma (IDC) and invasive lobular carcinoma (ILC), which are the most common subtypes. On the other hand, sarcomas are rare and arise from the myoepithelium of connective tissues within the breast, such as muscles, fat, and blood vessels [2]. The scope of this review is limited to carcinomas, and sarcomas will not be discussed in detail.

The staging and grading of breast cancer are crucial for determining prognosis and developing treatment protocols. Staging refers to the extent of cancer spread and is typically classified according to the TNM system, which assesses tumor size (T), lymph node involvement (N), and the presence of metastasis (M). Stages range from 0 (in situ, non-invasive cancer) to IV (Invasive cancer with distant metastasis) [1]. Grading, on the other hand, evaluates the appearance of cancer cells under the microscope and their rate of growth. The Nottingham grading system is commonly used, categorizing tumors into grades 1 (low grade, well-differentiated), 2 (intermediate grade, moderately differentiated), and 3 (high grade, poorly differentiated) [3]. Understanding the different subtypes of breast cancer at the molecular level is important to develop effective novel treatment strategies and improve patient outcomes. This review will delve into the molecular biological characteristics of breast cancer subtypes while uncovering racial differences.

## 2. Epidemiology and Risk Factors of Breast Cancer and Racial Divergence

In 2022, 2,308,897 new breast cancer incidences were reported worldwide making breast cancer as the leading cause of cancer in women and the second leading cause of all cancer incidents. It also accounts for the highest number of cancer-related deaths in women worldwide and in 157 countries, reporting 665,684 deaths in the same year. Breast cancer incidents were higher in transitioned countries; however, breast cancer-related deaths are higher in transitioning countries [4]. In the United States, when non-melanoma of the skin is excluded, breast cancer is the most diagnosed cancer in women. Followed by lung cancer, it is also the second leading cause of cancer-related deaths in women [5].

Development of breast cancer is linked to accumulations of mutations in critical regions of the genome such as regions involving cell proliferation and division, DNA repair, and programmed cell death. These mutations can be inherited/germline or spontaneous/somatic. Hence, etiological factors of breast cancer include both hereditary and nonhereditary factors [6]. Some of the non-hereditary breast cancer risk factors are modifiable and 30% of breast cancer cases are attributed to these factors. Modifiable risk factors include excess body weight, physical inactivity, and alcohol intake [5]. Other risk factors based on epidemiological studies include early age at menarche, later age at menopause, advanced age at first birth, fewer children, less breastfeeding, hormone-replacement therapy, and oral contraceptives [7,8]. Germline mutations related to breast cancer include *BRCA1*, *BRCA2*, *CHECk2*, *ATM*, *PALB2*, and *PTEN* [9]. However, about half of breast cancers are unrelated to known risk factors [10].

In terms of the racial divergence of breast cancer, African American women have a two- to three-fold higher risk of breast cancer (more likely to develop the disease over their lifetime) than women of other racial groups and have a 36% higher death rate than Caucasian women, after age adjustment, despite having similar incident rates [11,12]. African Americans also tend to have a younger age distribution and more advanced-stage disease [13]. These can be attributed to socio-economic factors such as access to health care, post-surgical care, and food habits as well as biological factors such as tumor microenvironment (TME) composition, genomic aberrations, and cytokine and chemokine secretion [11]. Women with African Ancestry (AA) have a higher incidence of triple-negative breast cancer (TNBC), which has the worst prognosis among breast cancer subtypes. A higher allosteric load in AA women could contribute to breast cancer disparity [13].

In certain populations, more prevalence of germline mutations related to breast cancer such as *BRCA1*, *BRCA2* is observed. In the general population, the frequency of *BRCA1* and *BRCA2* variants are 1 in 300, and 1 in 800, respectively. However, some of the *BRCA1/2* variants are found in Ashkenazi Jewish women at a 1 in 40 frequency [14]. Haplotypes that are inherited altogether can influence breast cancer susceptibility [15,16].

Furthermore, certain non-hereditary risk factors such as dietary patterns have ancestral influence. The traditional Mediterranean diet has been proven to provide protective effects against the risk of breast cancer, especially postmenopausal breast cancer [17,18].

## 3. The Current Paradigms of Breast Cancer Diagnosis and Treatment

### 3.1. Breast Cancer Diagnosis

Mammography, an X-ray-based imaging system, is widely used for screening breast cancer. Currently, high-resolution mammography is the gold standard reference for detecting primary breast tumors. Mammography aids in detecting the location and size of lesions through contrast and density abnormalities in the acquired images. However, small lesions are often missed, and a mammography has low sensitivity for dense breast tissue leading to false negative results [19,20,21,22]. Recent advancements have improved its diagnostic accuracy, particularly with digital breast tomosynthesis (DBT), which creates a 3D breast image [23]. Other derivatives of a mammography include full-field digital mammography (FFDM) and contrast-enhanced mammography (CEM) [24].

Currently, secondary imaging techniques include magnetic resonance imaging (MRI), ultrasound, and, in specific cases, PET/CT [25]. The most sensitive modality for breast cancer detection is MRI, which, due to its high cost, is used for breast cancer diagnosis of high-risk populations such as women with a family history of breast cancer [19,26,27]. In addition to secondary imaging, a recent study shows that an ultrasound is feasible as a primary tool for the early detection of breast cancer when mammography is not available [28]. The PET/CT is mostly useful for monitoring treatment response and detecting recurrence, and metastatic breast cancer [21,29].

While imaging techniques are invaluable for detecting and assessing breast abnormalities, they often cannot confirm malignancy. After revealing suspicious lesions, a biopsy is obtained for histopathological examination to confirm malignancy, determine the tumor subtype and develop a suitable treatment plan. A biopsy is a procedure where tissue is obtained from the patient for molecular, cytological, and histological analyses. There are the following three types of tissue biopsies used in breast cancer diagnostics: fine needle aspiration (FNA), core needle biopsy (CNB), and excisional biopsy [29]. The FNA collects some cells from the breast mass using a smaller solid needle which is usually 25-gauge. In CNB, a larger, hollow needle, which is generally about 14-gauge, is utilized to remove a cylindrical-shaped tissue core from the breast mass. It has been shown that the selection of the biopsy method affects the prognosis of the patients [30].

After obtaining the tissue biopsy, hematoxylin, and eosin (H&E) staining is used for primary diagnosis, followed by immunohistochemical (IHC) testing for hormone receptors (ER and PR), HER2 status, and KI67. Advances in IHC are achieved through automated IHC biomarker scoring with image analysis software [31]. It has been reported that H&E staining combined with machine learning can detect the ER status thus overcoming the limitations associated with IHC [32]. In addition, next-generation sequencing is currently employed to assess the presence of molecular alterations, including *PIK3CA* mutations and germline mutations in *BRCA1* and *BRCA2* [33].

In addition to tissue biopsy, liquid biopsies are emerging as potential diagnostics in breast cancer due to their superior characteristics such as noninvasiveness, achieving time-specific information and enhancing early detection possibilities. Cell-free DNA/RNA and circulating tumor cells have been explored as liquid biopsy markers [34].

### 3.2. Breast Cancer Treatment

Breast cancer treatments include surgery, radiotherapy, and systematic drugs [35,36]. A study comparing the quality of life after 4 common surgical breast cancer treatments- breast-conserving surgery (BCS), mastectomy, autologous breast reconstruction, and implant-based breast reconstruction found that patients who underwent a mastectomy have the lowest mean health-related quality of life [37].

Radiotherapy is often successfully used after BCS–whether whole breast, partial breast, tumor bed boost, or regional nodes, and after mastectomy–to reduce the risk of local recurrence. However, it has been reported that radiation therapy increases non-breast-cancer mortality [38].

Systemic drugs for breast cancer treatment encompass chemotherapy, endocrine therapy, targeted therapy, and immunotherapy [36]. Neoadjuvant chemotherapy (NACT) is used to downsize the tumor enabling operation on previously inoperable tumors. Furthermore, NACT enables surgeons to offer less complicated surgery or breast-conserving surgery (BCS) instead of mastectomy. The NACT does not cause postoperative complications [39]. Adjuvant chemotherapy has proven effective in improving disease-free survival, with certain drugs, such as the paclitaxel–carboplatin regimen, showing superior prognosis compared to standard anthracycline-based regimens in patients with TNBC [40].

While endocrine therapy is effective for hormone receptor-positive breast cancer, its side effects can significantly influence patients’ choice of treatment [41]. Immunotherapy has also become standard care for breast cancer, especially for TNBC [42,43]. Patients with biomarkers like PD–L1 expression and tumor-infiltrating lymphocytes have shown promising results indicating better responses to immunotherapy [44].

For patients with germline *BRCA* mutations, PARP inhibitors have been approved and offer a targeted treatment option [45]. Patients receiving HER2-targeted therapies should be closely monitored for cardiac toxicity, necessitating regular cardiac evaluations during treatment [46].

The combination of CDK4/6 inhibitors and endocrine therapy has become a prime treatment approach for patients with hormone receptor-positive (HR+)/HER2-negative (HER2-) advanced breast cancer. Three CDK4/6 inhibitors are currently approved for breast cancer treatment [37,47].

## 4. Histopathologic and Molecular Subtypes of Breast Cancer

### 4.1. Histopathologic Subtypes of Breast Cancer

Breast carcinoma is the uncontrolled growth of abnormal cells in epithelium layers of breast tissue. Histological variation exists in breast cancer, leading to multiple subtypes with different clinical implications. Most breast cancers arise either in the lobes (lobular carcinoma) or ducts (ductal carcinoma). Lobules are small milk-producing units and the ducts are the tubular structures that connect the lobules to the nipple [2,48,49]. However, some scientists identify that all breast carcinomas arise from the terminal duct lobular units which are the parts of the breast where the terminal ducts connect with the lobules [50,51,52,53].

Histologically, there are the following two major types of breast cancer: In situ and Invasive. When abnormal cells are confined to the layer of epithelial cells where they originated, it is considered in situ carcinoma. In situ is further divided into the following two categories: ductal carcinoma in situ (DCIS) and lobular carcinoma in situ (LCIS). Invasive carcinomas occur when abnormal cells spread out of the epithelium layer of initiation. Invasive breast carcinoma divides into several subtypes. Invasive ductal carcinoma (IDC) and Invasive lobular carcinoma (ILC) are the most common subtypes.

There are less frequent invasive subtypes such as medullary, apocrine, tubular, mucinous, metaplastic, cribriform, neuroendocrine, classic lobular, and pleomorphic breast cancer [1,54].

The DCIS is identified as stage 0 breast cancer and is a precursor to invasive breast cancer. Out of newly diagnosed breast cancer, 20% are DCIS. Architectural patterns classify DCIS into comedo, papillary, solid, cribriform, and micropapillary subtypes. Based on histopathologic nuclear features, DCIS can manifest across a spectrum, ranging from low-grade (I) to intermediate-grade (II) and high-grade (III) lesions. High-grade lesions are associated with a greater risk of developing into invasive ductal carcinoma (IDC) [55]. The LCIS is neither considered true breast cancer nor a precursor for Invasive breast cancer. It is only associated with an increased risk of invasive breast cancer [8]. However, some authors identify LCIS as a precursor for ILC [56,57,58,59]. The LCIS is categorized into the following three variants: classic LCIS, Pleomorphic LCIS, and florid LCIS [57,60].

The IDC is the most common histological subtype of breast cancer [55]. The IDC is composed of different morphological variants and clinical outcomes among individuals resulting in difficulties in treating appropriately. Tumor cells in IDC appear in different sizes and shapes, with prominent nucleoli and a high number of mitoses [54].

About 5–15% of diagnosed breast cancers are ILC. In the classic form of ILC, small tumor cells with little atypia in concentric uniform distribution can be observed. In pleomorphic ILC, cells appear to have apocrine gland cells, with hyperchromatic and off-center nuclei. Numerous mitotic figures are also visible [54,61].

Biology, clinical behavior, and prognosis of less frequent invasive breast carcinoma subtypes are diverse and not well understood. They are difficult to investigate due to their rarity, lack of interest, and funding [62]. The overlapping features with more common subtypes have made it difficult to diagnose the rare subtypes. In addition, most of the standard treatment protocols are formulated for more common subtypes which could affect treatment outcomes [63,64].

### 4.2. Molecular Subtypes of Breast Cancer

Even in the same histological subtypes, there is heterogeneity at the molecular level. The molecular classification of breast cancer into distinct subtypes has been a significant advancement in understanding the disease’s heterogeneity and guiding treatment strategies. The four major molecular subtypes of breast cancer are Luminal A, Luminal B, Basal-like (Triple Negative), and HER2-enriched. This classification is based on the expression pattern of hormone receptors (HR)-estrogen receptors (ER), progesterone receptors (PR)- and the human epidermal growth factor receptor 2 (HER2). Luminal A is the most common subtype of breast cancer (68% of all cases) characterized by HR+/HER2−. Luminal B is characterized by HR+/HER2+ and/or the presence of specific proteins indicative of rapidly dividing cells. Luminal B is about 10% of all breast cancers. Basal-like or Triple-negative breast cancers do not express HR and HER2 and are the most aggressive type of breast tumor. The TNBC is about 10–20% of all breast cancer patients. The HER2 enriched category is the least common category and is identified by HR−/HER2+ [1]. For a timeline of development of this classification, please refer to Zhang X, 2022 [65]. Some authors identify rare subtypes of normal-like and claudin-low as well [11].

Different molecular subtypes of breast cancer have different clinical behaviors and prognoses that are treated by various therapeutic regimens [66,67]. Both DCIS and IDC contain the above molecular subtypes; however, luminal B and HER2 enriched subtypes are more prevalent in DCIS compared to the IDC. The IDC lesions are much more likely to be luminal A phenotype [55,68]. The Basel subtype is prevalent in both high-grade DCIS and IDC. Furthermore, immune cells have been identified in different frequencies in different molecular subtypes of ductal carcinoma. In Luminal A, the proliferation marker, Ki67, is low, indicating a favorable prognosis, while the other three types have high Ki67 expression, indicating aggressive behavior [68].

The four molecular subtypes can be observed in ILC as well. However, there is a difference in the prevalence of each subtype compared to the IDC. Based on Immunohistochemical analysis and gene expression profiling, 85% of classic ILCs are Luminal A. Luminal B cancer appear less often, while HER2-enriched and basal-like subtypes are rare in ILC. The prevalence rate of different subtypes also depends on the stage of the ILC [69,70].

Characteristics of a molecular subtype vary based on the histological subtype of breast cancer. For example, a recent study shows that the basal-like subtype in ILC less frequently expresses basal markers (CK5/6, EGFR, and SOX10) compared to basal-like IDCs [71]. Yang et al. reported different overall survival (OS) in IDC vs. ILC in the same molecular subtype (HER2−) and ILC had worse OS in the subgroup compared to the IDC [72]. Molecular subtypes carry prognostic markers, and, in the clinic, assessment of the expression of hormone receptors and HER2 overexpression aids in determining the targeted therapies [73].

## 5. Histopathological and Molecular Progression Pattern of Breast Cancer

### 5.1. Histological Progression of Ductal Breast Carcinoma

Abnormal cells that are initiated at the duct can develop into atypical ductal hyperplasia (ADH) then into ductal carcinoma in situ (DCIS) and later into invasive ductal carcinoma (IDC). About two decades ago, the precursor lesion for ADH was mostly identified as flat epithelial atypia (FEA) [59,74,75,76]. Later with advancements in molecular and genetic evidence, histopathological findings, and clinical data, FEA appeared to have a lower risk of progressing to breast cancer [77,78,79,80]. Currently, the most accepted model suggests that Usual Ductal Hyperplasia (UDH) is a more promising precursor for ADH.

In several studies, when following up after diagnosis, 25–60% of untreated DCIS progress to IDC within 9–24 years. However, the progression mechanism is not yet precisely understood [55]. There are the following four accepted evolutionary models of DCIS progression to IDC: independent lineage model, convergent phenotype model, evolutionary bottleneck, and multiclonal invasion. The independent lineage model suggests that DCIS and IDC progress from different cancer-initiating cells of the same breast. The other three models propose that DCIS and IDC arise from common ancestors. According to the convergent phenotype model, DCIS with different genetic profiles evolves to form the IDC of a single phenotype. On the other hand, the evolutionary bottleneck model posits that out of the multiple DCIS clones with different genetic profiles, one clone with selected characteristics will evolve to IDC. Finally, the multiclonal invasion model suggests that different DCIS clones will progress and persist in IDC [81,82].

Another controversy related to breast cancer is the treatment of DCIS. Currently, all diagnosed DCIS are treated. However, many scientists agree that DCIS diagnosis is overtreated because not all DCIS will progress to IDC [82,83,84]. There is a pressing need for studies that will assist in distinguishing high-risk DCIS from low-risk DCIS to prevent the consequences of overtreatment [83,84].

### 5.2. Histological Progression of Lobular Breast Carcinoma

The model of lobular breast cancer progression is debated; some authors identify ALH and LCIS as precursor lesions and some do not, as previously stated. One published model of lobular breast cancer is as follows. During lobular breast cancer progression, normal epithelial cells of the breast will develop into atypical lobular hyperplasia (ALH). The ALH will proliferate and progress to Classic LCIS (CLCIS), which could further progress to Classic ILC, Alveolar ILC, Solid ILC, and Tubulo-lobular ILC. Pleomorphic LCIS (PLCIS) and Florid LCIS (FLCIS) can arise either from ALH or CLCIS. The PLCIS can develop into Pleomorphic ILC. The FLCIS can develop into all types of ILCs including classic ILC, alveolar ILC, solid ILC, Tubulo-lobular ILC, and Pleomorphic ILC [58].

### 5.3. Molecular Progression Pattern of Breast Cancer

Molecular changes accompany the histological progression of breast cancer [81]. The gene expression profile of breast cancer along the histopathological progression has been linked to validate the relationship among the progression stages [74]. In UDH, heterogeneous or mosaic patterns of high-molecular-weight cytokeratin such as CK 5/6, CK14, and 34βE12 are observed. Patchy and weak-to-moderate staining of ER is also a characteristic of UDH [85,86]. Original research related to UDH molecular biology has been scarce in the past several years. The lack of clinical relevance of this benign lesion could be a possible reason for limited research. However, researching UDH could increase understanding of breast cancer etiology, and progression patterns and develop noninvasive early detection and prevention methods.

The ADH are mostly ERα positive, and they tend to progress to ERα positive tumors. Estrogen signaling has been associated with the progression of ADH to invasive cancers [86]. Recent studies highlighting how ER-negative breast cancers evolve are scarce. The HER1 and IGF–1R are also increased in Atypical Hyperplasia [87]. In both ductal and lobular atypical hyperplasia, SFRP1 expression is decreased, and it is a significant regulator of the ADH transcriptional profile. However, ADH has higher E-cadherin expression levels compared to ALH [86]. The ADH lacks CK5/6 expression allowing the distinction between UDH and ADH [88]. Losing 16q and 17p, and gaining 1q, has been identified as a recurring alteration in ADH. Similar alterations could be observed in low-grade DCIS indicating the precursor and resulting product relationship [89]. Hotspot point mutations in PIK3CA are identified in both usual and atypical hyperplasia. This may suggest a potential role in these mutations in breast epithelial proliferation and atypical change. The ERBB2 (HER2) overexpression is observed in atypical hyperplasia and indicates an increased risk of subsequent breast cancer [90,91]. In contrast, some authors identified that ADH and low-grade DCIS are HER2 negative [92]. The TP53 mutation is observed in ADH, DCIS, and IDC in increasing order of abundance of mutation [55,90]. Research related to the molecular biology of ALH is limited, possibly because it is not established as a precursor lesion of invasive cancers.

**Figure 1:**
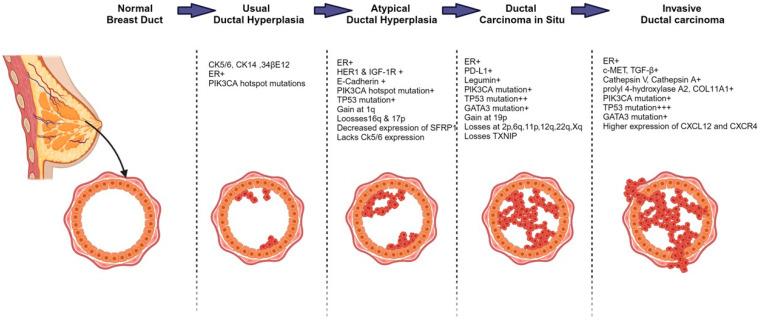
The histological progression model of ER+ ductal carcinoma and accompanying molecular and nuclear genetic alterations. Initiation of tumor cells from epithelium cells that are confined within the duct characterizes Atypical Ductal Hyperplasia. Ductal in situ carcinoma is the complete filling of the duct with tumor cells. When tumor cells escape the duct and spread, the invasive ductal carcinoma is formed. The molecular changes underlying each stage of progression are comprehensively depicted, highlighting key alterations that drive the transition from normal epithelium to invasive ductal carcinoma.

The expression of CK5/6 is associated with DCIS [93]. Around 87% of cases of DCIS are ER+ [94]. Furthermore, DCIS cells express PD–L1 which is an immune regulatory protein that modulates the immune system to prevent the detection and elimination of tumors by the immune system of the host [81]. When progressing to IDC, some of the HER2+ DCIS tend to lose HER2 [68]. Gains at 19p, and losses at 2p, 6q, 11p, 12q, 22q, and Xq, can be observed in DCIS and IDC [92]. The *PIK3CA* and *GATA3* mutations can also be observed in DCIS and IDC. However, IDC has more genetic instability, complexity, and a different transcriptomic profile compared to DCIS [55]. Since DCIS cells undergo a epithelial-to-mesenchymal transition (EMT) to migrate and be invasive, IDCs possess EMT markers such as c-MET and TGF-ß1. The IDC and adjacent DCIS show higher expressions of proteases that are responsible for extracellular matrix remodeling such as cathepsin V, cathepsin A, prolyl 4-hydroxylase A2, and COL11A1 which indicates the involvement of these proteins in DCIS progression. The DCIS gradually loses expression of thioredoxin interacting protein (TXNIP) which is a growth/tumor suppression protein and gains expression of legumain, which is a proliferation protein. The IDC also shows higher levels of certain chemokines like CXCL12 and its receptor CXCR4 which promote the migration of cells and immune evasion [81]. A graphical representation of the progression of ER+ ductal carcinoma, as discussed in this section, is shown in Figure 1.

## 6. The Pattern of Nuclear Genetic Alterations in Breast Cancer and Racial Differences

The genomic landscape and alterations that are associated with breast cancer development and progression are extensive and contribute to racial disparity. in the disease outcome. Alterations leading to breast cancer progression could be either germline or somatic. Understanding the nuclear genetic alterations in cancer is important in predicting tumor behavior, and treatment outcomes. It is also important in developing personalized medicine. Nuclear genetic alteration in cancer cells could be single nucleotide variants, short insertions/deletions, copy number variations, and fusions [65,95,96]. Based on a recent study, the prevalence of the top 10 most mutated genes in all cancers across the US population are as follows: *TP53* 34.5%, *P1K3CA* 13.5%, *LRP1B* 13.1%, *KRAS* 10.5%, *APC* 10.1%, *FAT4* 9.5%, *KMT2D* 9.2%, *KMT2C* 9.1%, *BRAF* 7.6%, and *ARDIA* 7% [97]. Out of these 10 genes, *TP53* was found in breast carcinoma at a frequency of 30% [98], *P1K3CA* at 20–40% [99], and *KMT2C* at 6–9% [100].

Mutations at *PI3KCA* are frequent in ER+ tumors (Luminal). Out of diagnosed luminal tumors, close to 40% carry mutations in *P13KCA*. A recent study suggests *ARID1A* mutation coexists in certain *P13KCA* mutated luminal cancers and they are linked to immunity-related signaling pathways [101]. The *PIK3CA* mutations are more common in patients with European ancestry compared to patients with African ancestry [102]. Luminal B cancer has a lower frequency of *PIK3CA* mutations compared to Luminal A [72]. The HER-2 enriched had an even lower frequency than Luminal B. Basal-like (triple negative) and has the lowest prevalence of *PIK3CA* mutations [103].

The *TP53* mutations are associated with aggressive tumor characteristics and clinical relevance varies across subtypes. The *TP53* mutations are more prevalent in HER2-positive and triple-negative breast tumors (TNBC), while the incidence is low in luminal A tumors [104]. In a study conducted in 7226 Chinese women, the frequency of p53 protein expression also varied by subtype [105]. The *TP53* mutation is more prevalent in African Americans compared to European Americans [106]. This racial difference in the TP53 mutation contributes to the higher tumor recurrence in patients with African ancestry.

The *KMT2C* mutations were linked with older breast cancer patients and the most frequently reported in HR+/HER- breast cancer [107]. The *MAP3K1, FOXA1*, and *GATA3* are also frequently mutated in ER+/luminal breast tumors [92,108].

Germline pathogenic variants in CDH1 are associated with an increased risk of lobular breast cancer [109,110]. It is also associated with hereditary diffuse gastric cancer [111].

There are over 800 genes linked with TNBC pathogenicity in terms of differential expression or mutations [95]. As identified by many NGS studies, recurrent pathogenic alterations in TNBC are TP53 mutations, immune checkpoint response genes, and aberrations in the *PIK3CA* and DNA repair pathways [112]. An investigation to determine single nucleotide polymorphisms in genes linked with TNBC identified *BRCA1, BRCA2, EGFR, PIK3CA, PTEN,* and *TP53* as the most extensively studied genes, each showing variations reported in over ten research studies [95]. It has been reported that there are no significant differences in the mutational profiles of TNBC tumors between African American and Caucasian women, suggesting that the nuclear gene mutation landscape is not associated with the higher prevalence of TNBC in African Americans. Table 1 represents a summary of the nuclear genetic signature of breast cancer subtypes.

## 7. Mitochondrial Genetic Heterogeneity in Breast Cancer Subtypes and Racial Divergence

Mitochondria play a crucial role in energy production and are often called the cell’s powerhouses. It also has an important role in cellular metabolism, apoptosis, and maintaining calcium and redox homeostasis. They communicate with other organelles such as the endoplasmic reticulum and lysosomes [119,120]. Mitochondrial functions are regulated by both mitochondrial and nuclear DNA. It has been well recognized that mitochondria play a crucial role in cancer development and progression by altering energy metabolism to support cell growth, impacting tumorigenesis, and therapy response. Cancer cells accompany mitochondrial DNA (mtDNA) abnormalities which alter energy metabolism in favor of cancer cell progression [121,122]. It has been reported that depletion of mtDNA resulted in diminished or no tumorigenesis which can be restored after reintroducing mtDNA [123].

**Figure 2:**
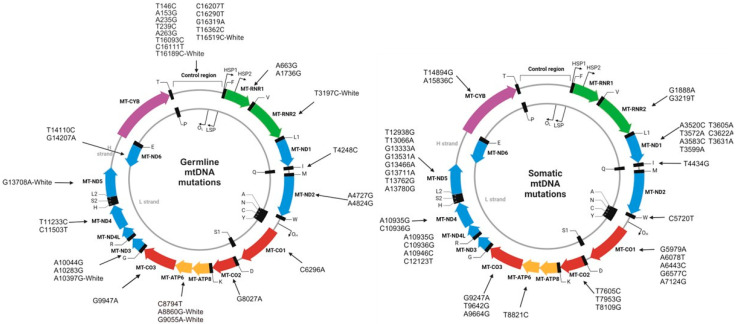
Germline and somatic mitochondrial DNA alterations/polymorphisms associated with breast cancer. The images were adapted from Biorender.com and modified as necessary. “-White” represents the mutations prevalent predominantly in the White American populations. (**Left)**: Represents germline mtDNA single nucleotide mutations/polymorphisms associated with breast cancer. Mutations were reported in the control, RNR1, RNR2, tRNA for isoleucine (I), *ND2, CO1, CO2*, *ATP6, CO3, ND3, ND4, ND5,* and *ND6* regions. (**Right)**: Represents somatic mtDNA single nucleotide mutations associated with breast cancer. Mutations are reported in the RNR2, *ND1*, tRNA for isoleucine (I), tRNA for tryptophan [W], *CO1*, *CO2*, *ATP6*, *CO3*, *ND4*, *ND5*, and *CYTB* regions. HSP: H strand promoter; LSP: Light strand promoter; ND: NADH dehydrogenase; CYTB: Cytochrome B; CO: Cytochrome c oxidase; ATP6: ATP synthase F0 subunit 6; ATP8: ATP synthase F0 subunit 8; MT: Mitochondrial; RC: Respiratory complex.

Building on the crucial role of mtDNA in cancer, studies have identified specific mtDNA variants, associated with an increased risk of breast cancer. These variants associated with breast cancers are mostly single nucleotide variants rather than small insertions or deletions [124]. Certain germline mutations/polymorphisms in mtDNA have been associated with breast cancer risk. For example, T to C substitution at np 16189 is associated with susceptibility to breast cancer development [125]. In addition, 16093T>C/T16093C mutation increases 67% risk of developing breast cancer. Figure 2 (left) represents commonly observed mtDNA germline mutations based on references [125,126,127].

Furthermore, it has been described that somatic variation of mitochondrial DNA mutation and copy number exist in breast tumors. However, the link between these variants and the phenotypical behavior of cancer and clinical outcome has not been very well understood yet. Several studies report lower mtDNA content in breast tumor tissue than in adjacent normal mammary epithelium. In addition, lower mtDNA content has been reported as the worst prognosis [124].

A recent study conducted using 92 matched primary breast tumors and peripheral blood samples reported somatic mtDNA mutations in 73.9% of breast tumors predominantly in the coding region. However, no significant correlation was found between mtDNA mutational burden and overall survival [126]. In contrast, another study reported a higher prevalence of mutations at the noncoding region D-Loop. Out of 27 somatic mtDNA mutations identified, 22 are at D-Loop [128]. In addition, D-Loop mutation profiles of ovarian and breast cancers were found to be similar [129].

Some somatic mutations in breast tumor mtDNA have been detected in matched nipple aspirate fluid [130]. Somatic mutations detected in primary tumors have also been detected in metastatic lymph node [131]. Figure 2 (right) illustrates several somatic mtDNA mutations reported in the literature [34,126,128,132].

Another recent study examined mtDNA alterations (both mutation and copy number) in 32 TNBC patients [132]. A panel of 11 frequently occurring and hotspot mtDNA mutations were identified in this study. Notably, TNBC patients with African ancestry had a higher mtDNA mutation load compared to the patients with European ancestry. Interestingly, 82% of the TNBC-tumor-derived hotspot mtDNA mutations were readily detectable in matched serum-extracellular vesicles (EVs) of these patients. The 82% of the TNBC-tumor-derived hotspot mtDNA mutations were readily detectable.

**Figure 3:**
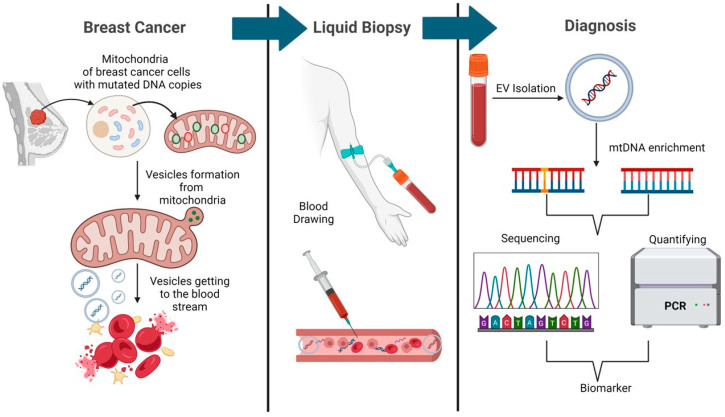
Strategy for the development of minimally invasive mtDNA based biomarker tools for early detection of breast cancer. The mitochondria derived extracellular vesicles (EVs) harboring mutated mtDNA enter the bloodstream. Blood is drawn from the patient and processed to isolate EVs, which are then subjected to mtDNA enrichment. The enriched mtDNA is analyzed through mitochondrial whole genome sequencing and quantified using PCR to identify mtDNA based biomarkers of breast cancer. This noninvasive approach could facilitate early detection, monitoring, and therapeutic planning in breast cancer patients.

The EVs obtained from these nine patients’ sera also had an exclusive abundance of mtDNA content compared to EV–mtDNA from cancer free subjects [132]. Similar to mtDNA, an enrichment of cardiolipin content (an exclusive inner mitochondrial membrane lipid) was also evident in the sera-derived EV of these nine patients compared to the EVs obtained from cancer-free individuals. This study demonstrates the potential of mtDNA alteration detection in the circulating EVs as a minimally invasive biomarker tool for early aggressive breast cancer detection, monitoring, and risk assessment [132]. A comprehensive analysis of mtDNA alterations in tumors and body fluid samples of all the major molecular subtypes of breast cancer will be useful in developing liquid biomarkers for the early detection of lethal lesions, their accurate monitoring, and therapeutic planning (Figure 3).

## 8. Epigenetic Alteration Signatures of Breast Cancer Subtypes in Racially Disparate Populations

Epigenetics refers to inheritable changes in gene expression that do not involve alterations to the underlying DNA sequence. Mechanisms of epigenetic gene regulations involve DNA methylation, histone modification (methylation, acetylation, phosphorylation), and non-coding RNAs. Diet, exposure to pharmaceutical and toxic chemicals, stress, exercise, etc. affect the epigenetic landscape [133]. In addition to genomic alterations as discussed previously in this article, the epigenetic landscape contributes to breast cancer development and progression as well as its heterogeneity [134]. BRCA1 promoter hypermethylation is linked to higher risk of breast cancer and aggressiveness. Hypermethylation is observed in malignant breast tumors and normal adjacent tissues compared to benign breast lesions [135].

Epigenetic regulators acquire modification leading to an altered epigenome. A 2023 study by Liu et al., identifies FOXA1, a transcription factor involved in epigenetic reprogramming crucial for breast cancer progression, implements its oncogenic function via O-linked β-N-acetylglucosamine modification (O-GlcNAcylation) of FOXA1. This modification of FOXA1 promotes breast cancer metastasis by setting up the transcription of numerous metastasis regulators [136]. Flavonoids have been identified as an influencer of the epigenetic mechanisms of breast cancer. Flavonoids such as epigallocatechin, genistein, and resveratrol inhibit DNA methyltransferase and altered chromatin modification in breast cancer which can induce different tumor suppressor gene expressions that may contribute, to decreasing breast cancer progression and metastasis [137].

Differential epigenetic alterations have been observed among subtypes of breast cancer. Variation in methylation levels of histone 3, lysine 27 (H3K27me) is associated with gene repression and serves as a hallmark of breast cancer transformation. Higher trimethylation of H3K27 (H3K27me3) has been linked with better BC outcomes. H3K27Me3 was reported at higher levels in luminal A, HER2-enriched, and normal-like tumors compared to triple-negative, and luminal B subtypes [138]. Hypermethylation in BRCA1, was highest in the HER2+ subtype while ER+/HER2- had the lowest hypermethylation [135].

Variations in epigenetic alteration patterns have been observed among African American and European Americans as well. More differentially methylated loci (DML) tumors were identified in black women than in white women. Some of the DMLs in noncoding regions are associated with altered gene expression supporting their potential roles in contributing to breast cancer differences between the two races [139]. Another study reported 1115 differentially methylated sites among younger African American females with TNBC compared to older African Americans as well as White women of all ages [140].

## 9. Microbiome Alteration Pattern in Breast Cancer Subtypes and Their Racial Distribution

The human microbiome consists of the genomes of various microbes including bacteria, fungi, phages, archaea, and viruses which coexist in the sites of the human body such as the gut, skin, lung, vagina, and oral cavity. Different sites have different compositions. Research has shown that microbiomes are important in immunity and other health functions such as nutrient extraction, metabolism, and biosynthesis of bioactive molecules. Dysbiosis of microbiomes is associated with various diseases including cancer. Microbiomes have significance in carcinogenesis by influencing host cell proliferation, immune system activity, and metabolism via several mechanisms [141]. It has been identified that disruption of the natural host–microbiome interaction promotes breast cancer tumorigenesis via altering immune system functioning, estrogen levels, and bacterial metabolites. The gut and breast microbiome play a crucial role in breast cancer development, progression, and treatment outcomes [142,143,144,145,146]. Breast cancer patients exhibit a notable change in gut microbial composition compared to healthy women, characterized by variations in the relative abundance of specific microbial species and families [108].

Distinct microbiome profiles have been identified by various research of breast cancer tissue, noting variations in microbial diversity and abundance across different molecular subtypes, breast tissue type (tumor, tumor-adjacent normal, high-risk, healthy control), cancer stage, grade, histologic subtype, receptor status, lymph vascular invasion, or node-positive status [10,147].

A comprehensive study was performed in 2021 using the pan-pathogen microarray strategy to determine the breast cancer microbiome of the following four different subtypes: (1) ER and/or PR-positive, and HER2-negative (ER); (2) triple-positive that are ER, PR, and HER2-positive (TP); (3) HER2-positive and ER and PR-Negative (HR) (4) ER, PR, and HER2-negative (TN). The most diverse microbiome was in the ER while the TN had the least diverse microbiome. The TN microbiome was significantly different from the rest of the subtypes as fewer microbial agents were detected, a significant prevalence of Aggregatibacter was observed, and Plagiorchis and Trichostrongylus were detected. The ER had the greatest number of bacterial signatures which were mostly Proteobacteria. The ER’s fungal, viral, and parasitic signatures were also highest among subtypes. The microbiome of TP showed greater similarity to ER. Unique microorganisms were present in each subtype which could specifically distinguish subtypes [148].

Gut microbiomes and their association with breast cancer subtypes have also been researched. A higher abundance of the genus Sellimonas and Adlercreutziain in the gut is causally linked to an increased and decreased risk of ER+ breast cancer, respectively. In HER2+ breast cancer, an elevated abundance of genus Erysipelatoclostridium was associated with an increased risk of breast cancer while genus Ruminococcus2 is linked to a decreased risk of breast cancer [149].

**Figure 4:**
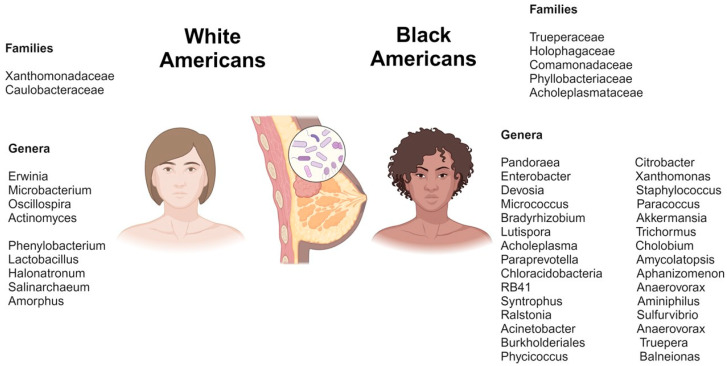
This figure demonstrates the most prevalent microbial families and genera found in breast tumors of White American and Black American women. Microbial composition varies between these populations, as shown by the distinct genera and families listed for each. Data are based on studies exploring microbiome diversity in breast cancer across racial groups.

Furthermore, research has also identified significant differences in the breast microbiota between racial groups [150]. Non-Hispanic Black (NHB) women with breast cancer tend to have different microbial profiles compared to Non-Hispanic White (NHW) women [151,152]. The microbiota composition of TNBC tissue of NHB showed a significantly reduced diversity as compared to adjacent normal tissue. In contrast, the tumors from NHW showed higher diversity in tumor tissue compared to normal tissue [152]. Phylum Actinobacteria (*p* = 0.03) and the unclassified genus of Bradyrhizobiaceae (*p* = 0.03) were underrepresented in TNBC from black women, highlighting the microbiome as a potential racial-specific biomarker [151]. Another large-scale study compared the bacterial community composition of 1018 primary tumors from Asian (*n* = 65), Black (*n* = 257), and white (*n* = 696) women. The microbiota of tumor tissue from Asian women did not show significant differences from both black and white people based on alpha and beta diversity measures. However, microbiota in breast tumors from Black and white women revealed significant differences in both alpha- and beta-diversity measures [151]. The diversity of microbiota in breast cancer tissue from White and Black women is depicted in Figure 4, as reported by these studies. In this figure, only consistent families or genera across studies are included, while contradictory ones are excluded. On the other hand, studies determining the racial differences in gut microbiota of breast cancer patients are warranted.

## 10. Future Perspective

While advancements in breast cancer diagnosis and treatment have significantly reduced mortality rates, there several critical areas for future research remain to improve the long-term disease-free survival of the patients. Comprehensive and in-depth molecular profiling, understanding the environmental interplay and formulation of strategies to minimize therapeutic side-effects will be crucial in providing a longer disease-free healthy life to the patients with diverse racial and ethnic backgrounds. In concert, the development of reliable noninvasive or minimally invasive tools for the early detection of lethal lesions in various molecular subtypes from racially disparate populations, their accurate monitoring, and therapeutic planning would help improve the overall survival of the patients. With the advent of single cell sequencing, spatial genomics, CRISPR–CAS9, non-CRISPR based genome editing approaches and combined machine learning techniques, the next few decades will certainly refine these areas of breast cancer research and existing racial disparities, which will ultimately improve the overall survival and quality of life of the patients.

## 11. Conclusions

Breast cancer encompasses a complex landscape of histopathological and molecular subtypes, each with distinct clinical behaviors and prognostic implications. The histologic and molecular heterogeneity of breast cancer underscores the need for precise classification systems and subtyping to predict prognosis and make treatment decisions. While substantial progress has been made in our understanding of breast cancer in general, critical gaps remain in areas such as risk assessment, early detection of certain molecular subtypes (e.g., TNBC), accurate surveillance and management of therapeutics and therapy induced side effects. Moreover, racial differences in breast cancer incidence, molecular and histological progression, and outcomes necessitate a deeper exploration of underlying biological and socio-economic factors. Development of race-specific animal or organoid model systems to unravel the enormously complex interplay in the milieu of tumor microenvironment and heterogeneity will also be necessary.

## Figures and Tables

**Table 1:** Nuclear genetic alteration signature in various molecular subtypes of breast carcinomas.

Breast Cancer Subtypes	Genetic Signature
Luminal A	Highest frequency of *PIK3CA* mutation among the 4 subtypes [113]
	*TP53* mutations are the second most common mutation in Luminal A after *PIK3CA* but occur less frequently compared to the other subtypes [98,114]
	Mutations in *CDH1* [114]
	High expression levels of *GATA3* [115]
	Higher frequency of *GATA3* mutation compared to luminal B [116]
	Mutations in *MAP3K1* [108]
Luminal B	Mutation in *PIK3CA* [85]
	Mutations in *GATA3* [116]
	Mutations in *MAP3K1* [108]
	Mutation in *TP53* [98]
HER2 enriched	Mutations in *TP53* [117]
	Mutations in *PIK3CA* [117]
	*CDH1*, *MAP3K1*, *GATA3*, and *ERBB2* mutations are detected with low frequency [117]
Triple-negative breast cancer	The highest frequency of *TP53* mutation among the 4 subtypes [98]
	*PIK3CA* mutations are the second most common mutation in TNBC after *TP53* but occur less frequently compared to other subtype types [113,118]
	*PTEN*, *KMT2C*, and *RB1* mutations are detected with low frequency [118]
